# Dr. Pierre Desnoyers Peltier

**Published:** 1906-09

**Authors:** 


Dr. Pierre Desnoyers Peltier, of Collinsville, Conn., died at his home July 21, 1906, of angina pectoris, aged 70 years. Dr. Peltier graduated in medicine from the University of Buffalo, with the class of 1860, was commissioned Assistant Surgeon of the One Hundred and Twenty-Sixth Regiment New York Infantry August 11, 1862, and resigned November 5, 1863. He was a man of accomplishments and took high rank in his profession.

